# A Meta-Analysis of the Existing Knowledge of Immunoreactivity against Hepatitis C Virus (HCV)

**DOI:** 10.1371/journal.pone.0038028

**Published:** 2012-05-31

**Authors:** Yohan Kim, Kerrie Vaughan, Jason Greenbaum, Bjoern Peters, Mansun Law, Alessandro Sette

**Affiliations:** 1 The Immune Epitope Database (IEDB), La Jolla Institute for Allergy and Immunology, La Jolla, California, United States of America; 2 Department of Immunology and Microbial Science, The Scripps Research Institute, La Jolla, California, United States of America; KAIST, Graduate School of Medical Science & Engineering, Republic of Korea

## Abstract

Approximately 3% of the world population is infected by HCV, which represents a major global health challenge. Almost 400 different scientific reports present immunological data related to T cell and antibody epitopes derived from HCV literature. Analysis of all HCV-related epitope hosted in the Immune Epitope Database (IEDB), a repository of freely accessible immune epitope data, revealed more than 1500 and 1900 distinct T cell and antibody epitopes, respectively. The inventory of all data revealed specific trends in terms of the host and the HCV genotypes from which sequences were derived. Upon further analysis we found that this large number of epitopes reflects overlapping structures, and homologous sequences derived from different HCV isolates. To access and visualize this information we developed a novel strategy that assembles large sets of epitope data, maps them onto reference genomes and displays the frequency of positive responses. Compilation of the HCV immune reactivity from hundreds of different studies, revealed a complex and thorough picture of HCV immune epitope data to date. The results pinpoint areas of more intense reactivity or research activities at the level of antibody, CD4 and CD8 responses for each of the individual HCV proteins. In general, the areas targeted by the different effector immune functions were distinct and antibody reactivity was positively correlated with hydrophilicity, while T cell reactivity correlated with hydrophobicity. At the sequence level, epitopes frequently recognized by both T cell and B cell correlated with low variability, and our analysis thus highlighted areas of potential interest for practical applications. The human reactivity was further analyzed to pinpoint differential patterns of reactivity associated with acute versus chronic infection, to reveal the apparent impact of glycosylation on T cell, but not antibody responses, and to highlight a paucity of studies involved antibody epitopes associated with virus neutralization.

## Introduction

With an estimated 130 to 170 million infections reported worldwide (∼3% of the world population), hepatitis C virus (HCV) represents a major global health challenge [1]. Accordingly, HCV immune reactivity has been the focus of intense investigation, to guide the efforts aimed at the development of an HCV vaccine, to probe HCV associated immunopathology, and as a tool to evaluate different vaccine candidates. As a result, a large amount of immunological data currently exists in the HCV literature, and a significant portion of this includes studies defining T cell and antibody epitopes.

The Immune Epitope Database and Analysis Resource (IEDB) provides the scientific community with a repository of freely accessible immune epitope data (www.immuneepitope.org). It contains data curated from published literature, and data submitted by NIAID's high-throughput epitope discovery projects, relating to antibody and T cell data for human, non-human primate, and rodent hosts, as well as a number of other animal species, and encompasses epitopes associated with all infectious diseases, autoimmunity, transplantation and allergy. Thus, the IEDB provides a unique resource to inventory and analyze immunological data for a given pathogen or disease. To date, we have performed a number of such meta-analyses of different human infectious agents, including influenza A, *M. tuberculosis*, Anthrax and Botulinum toxins, Plasmodium parasites and flaviviruses [2]–[6]. These analyses provide an overview of the current state of immunological data for a respective disease and highlight specific trends and identify areas in need of further experimentation. Furthermore, these meta-analyses are also meant to increase awareness of the information contained in the IEDB and solicit feedback to further improve the IEDB's usefulness.

In approaching a meta-analysis of HCV-related epitope data we were challenged by the large amount of data available. Since over 3,000 different epitopes, corresponding to largely overlapping or closely related homologous structures have been described, we developed a novel approach whereby immunoreactivity is assessed by mapping epitopes onto reference genomes and calculating aggregate frequency of positive responses.

The compilation and visualization of the HCV immune reactivity from nearly 400 different studies, painted an unprecedented and exhaustive picture of the efforts of the scientific community. The results provide a wealth of information inventoried for the scientific community, and revealed general features of HCV immune responses. At the same time the analysis identified specific knowledge gaps that represent areas for further study.

## Methods

### Targeted Data and Query

The complete epitope dataset was retrieved in January 2012 by performing queries from the IEDB homepage search interface (http://iedb.org) by specifying “HCV” as source organism and selecting the “T cell response” and “B cell response” choices offered in the IEDB webpage as Immune recognition context. MHC binding and MHC ligand elution data was excluded. This query will retrieve all available data in the IEDB for antibody and T cell epitopes associated with hepatitis C virus (all genotypes, subtypes and isolates) in human and nonhuman (animal models) hosts. The IEDB data are derived from the peer-reviewed literature indexed in PubMed. To be included in the IEDB, epitopes have to be mapped experimentally to a region of 50 residues or smaller. The IEDB captures epitopes and related data as defined in the literature and thus includes minimal/optimal epitopes (8–15 residues), larger less well-defined regions (16–50 residues), and key epitope residues identified as being involved in binding (1–2 residues). The IEDB curation process takes into account the fact that some residues may be important for protein folding instead of binding, and only studies providing controls addressing this issue are curated in the database. Negative structures (defined as structures for which only negative data has been reported) are also captured in the IEDB and have been included in this analysis. Additional detailed curation criteria can be found in [7]. Additional queries were performed to select subsets of HCV data using the “T cell search” and “B cell search” functions from the IEDB website, and specifying additional criteria to those mentioned above, such as response phenotype, host organism, HCV genotype or assay type. Results from each query were exported as Excel files and further analyzed in that format to generate particular tables and figures.

### Computational Methods

Each HCV immune epitope was mapped to a position in the polyprotein sequence of HCV H77, using a multiple sequence alignment (MSA) of HCV sequences. The use of a MSA permitted mapping epitopes that varied considerably from the HCV H77 strain. The MSA consisted of 10 HCV polyproteins; covering genotypes from 1 to 7 and 3 additional polyproteins from subtypes 1b and 2b to account for large sequence variability within genotypes 1 and 2 (**Table 1**). To assign a position to a peptide epitope, all peptides were mapped to each polyprotein and the best matching position was kept. Mapping was considered successful if a match with at least 50% sequence similarity was found. Second, all peptide matched positions were re-numbered with respect to the H77 polyprotein based on the MSA. A match was excluded if it was mapped to a gapped region in the reference sequence. Using this approach, 98.7% of HCV peptides were mapped onto the H77 sequence. For T cell peptides, 18 of the total 3,378 peptides were excluded. For antibody, 63 of the total 3,075 were excluded. When manually examining the reasons for peptide exclusion, we found that several were mapped to the core frame shift protein (F protein), represented consensus sequences for which no natural source can be identified, or from strains of naturally-infected subjects for which no sequence has been published to date. Therefore, we did not attempt to add additional strains to the MSA to further increase the mapping success rate.

**Table 1:** A list of HCV strains used for mapping peptides to polyproteins.

Genotype	Isolate/strain/serogroup	NCBI GI
1a	H77	22129793
1b	China	48237634
1b	JT	221615
2a	JFH-1	13122262
2b	HC-J8	221609
3a	Type V-D	633202
4a	ED43	2252490
5a	EUH1480	2462304
6a	6a33	57791994
7	QC69	124302095

### Correlation of response frequency with hydrophilicity and entropy

To measure sequence variability, entropy as a function of position in the polyprotein was calculated. Higher entropy indicates greater sequence variability. For the entropy calculation, a web tool provided by the HCV database at Los Alamos National Laboratory was used: http://hcv.lanl.gov/content/sequence/ENTROPY/entropy_one.html. The tool takes an MSA as input, and the MSA ‘Web alignment 2008’ provided by the HCV database was used: http://hcv.lanl.gov/content/sequence/NEWALIGN/align.html. The MSA consists of 471 representative HCV polyproteins from strains belonging to genotypes 1 to 6.

Hydrophilicity values were calculated, utilizing a tool housed on the IEDB webpage that utilizes the amino acid scale by Parker et al. [8] to plot average local hydrophilicity as a function of position. For this calculation a window size of 15 residues was used.

## Results

### An inventory of HCV immune epitope data in the IEDB

The IEDB contains data related to all epitopes from infectious diseases, allergy, autoimmunity and transplantation. As such it also contains HCV related data, as part of the general database. A search of the IEDB for HCV epitopes returned a total of 419 peer-reviewed references, 266 of which were related to T cell data and 174 of which were related to antibody data (**Table 2**). These references described a total of 6,202 unique molecular structures tested for immune reactivity, including structures only associated with negative data. Of the 3,444 structures associated with positive assays (from here after referred to as epitopes), 1,573 were identified in the context of T cell reactivity and 1,973 in the context of antibody reactivity.

**Table 2:** Summary of immune epitope data for HCV.

	References	Total Structures	Epitopes
B and T cell (All)	419	6,202	3,444
T cell	266	3,378	1,573
Antibody	174	3,097	1,973

The data represent all antibody and T cell epitopes reported for all HCV genotypes in all hosts. MHC binding data has been excluded. Non-peptidic epitopes and MHC ligand elution structures have not been described for HCV to date. ‘References’ denotes the number of individual peer-reviewed papers reporting HCV epitope data. ‘Total structures’ include positive and negative peptides. ‘Epitopes’ represents all positive structures. Clustering was performed using default 80% sequence homology of positive epitopes using IEDB Search interface. Note: total numbers for individual T cell and antibody responses may be more than the number of total reported epitopes because many structures are reported as both T and antibody.

**Figure 1:**
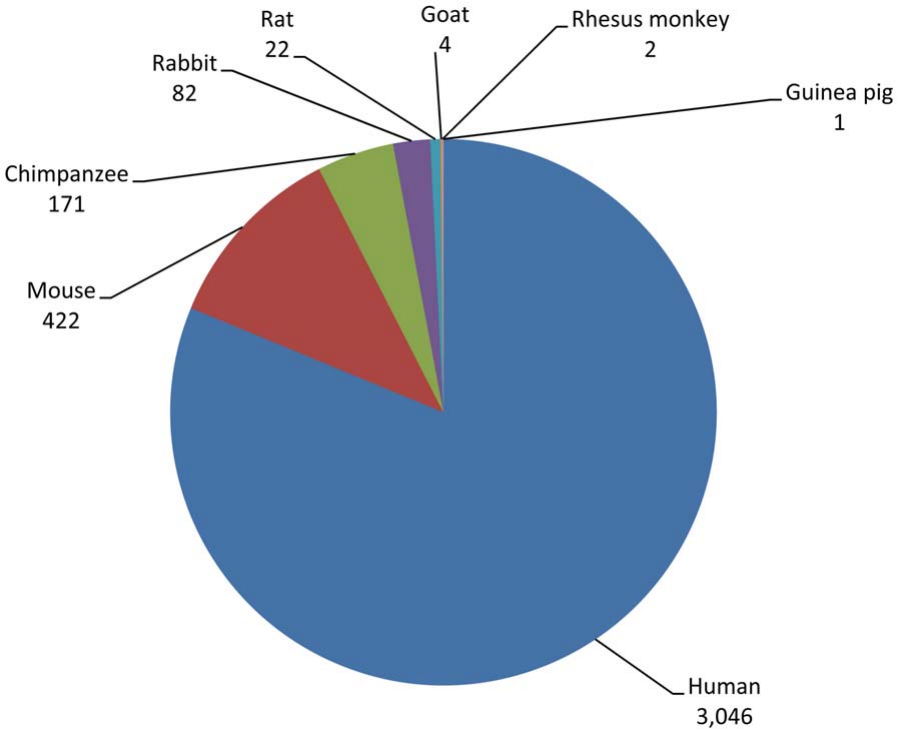
Epitope distribution among host species. Data represent the number of epitopes identified (in parentheses) for each host species reported to date.

HCV-derived epitopes have been identified in numerous species, including humans, chimpanzees, rhesus macaques, goats, guinea pigs, rabbits, rats and mice. **Figure 1** represents a breakdown of the current data with respect to the total number of epitopes per species. As expected, the vast majority of epitopes has been defined in human infection (3,046), and mice (422), while chimpanzees, a genetically relevant and immunologically parallel host represent only ∼5% of the data (171). The large number of murine epitopes was surprising in light of the fact that mouse is not a natural host for HCV. However this can be rationalized since mice are popular for raising mAbs and testing antigen immunogenicity. Of the more than 400 epitopes defined in mice, 75 were derived from HLA transgenic mouse strains (data not shown). Because of the relevancy of chimpanzees as a model of infection and disease, we also examined whether Patr- and HLA-restricted epitopes were located in the same regions. We found that chimp CD4/class II responses define 16 epitopes in shared antigenic regions, primarily NS3 and NS5a, whereas CD8/class I responses define 76 epitopes in shared regions across the polyprotein (data not shown).

HCV is officially classified into several genotypes, further categorized into subtypes and strains, based on genomic sequence heterogeneity. Genotypes 1–3 have a global distribution, with 1a and 1b accounting for ∼60% of infections worldwide [9]. **Table 3** provides a breakdown of all HCV epitopes reported in the IEDB by genotype. The vast majority of epitopes have been reported from genotypes 1a and 1b (1,016 and 862, respectively), which may reflect the overall prevalence of these genotypes. However it should also be considered that these number may be skewed by major research/sequencing effort in N America/Japan/Europe, rather than actual global prevalence. The selection of H77 as a prototypic strain, and drug resistance by genotype 1 viruses, has also steered research towards genotype1. For example, in Egypt approximately half million new infections/year are mostly associated with genotype 4, yet few research focuses on this genotype.

**Table 3:** HCV epitope distribution by genotype

	Total Positive	T cell	CD4/Class II	CD8/Class I	Antibody
**HCV genotype unspecified**	1,110	570	291	238	623
**Genotype 1**					
subtype unspecified	20	18	0	15	2
1a	1,016	670	267	257	349
1b	862	236	83	140	639
**Genotype 2**					
subtype unspecified	41	1	0	1	40
2a	112	25	1	24	88
2b	104	2	0	2	102
**Genotype 3** (all subtypes)	166	40	5	32	128
**Genotype4/5/6** (all subtypes)	13	11	2	9	2

The total number of epitopes reported for each genotype is presented. These data are then broken down by response phenotype: all T cell, CD4/class II, CD8/Class I and antibody. All reported genotypes are shown here; not all HCV genotypes have been studied. In some cases, genotypes were reported in the literature as simply ‘genotype 1’ or ‘genotype 2’ with no further specification provided. In other cases, no specific genotype was given and the authors simply referred to the epitope's source as HCV; it is these records that are presented with the qualifier ‘unspecified.’ For genotypes 3, 4, 5 and 6, data were bulked together (all subtypes), due to the small number of epitopes reported for each. The assignment of epitope genotype is made based on the assayed immunogen or antigen when these are indicated in the reference.

Somewhat surprisingly, reactivity against 1a has mostly been defined in T cells (670) with a smaller number being defined for antibodies (349). By contrast, the vast majority of 1b epitopes have been identified for antibody responses (639) compared to T cell (236). There have been a total of 112 epitopes identified from genotype 2a and 104 for 2b, the majority of which are antibody epitopes. Far fewer epitopes have been reported for genotypes 4, 5 and 6 (13). Finally, it should be noted that a very large number of epitopes (1,110) has been reported from unspecified ‘HCV’, derived from studies using human subjects for whom no HCV genotyping was reported.

### Mapping epitope data onto reference HCV genomes

Since the entire polyprotein consists of approximately 3000 amino acids, a significant level of redundancy and overlap must exist in the 3,444 “epitopes” reported in the literature. Reported epitopes may be closely related to each other because of sequence variation associated with different HCV strains and serotypes. Additional overlap results from different laboratories and experiments utilizing distinct, but largely overlapping peptides. Furthermore, little consistency exists between different reports regarding naming of proteins, and numbering of residue positions. Thus, to comprehensively analyze HCV related data, we developed an approach to organize the data in a consistent format.

For this purpose, all unique molecular structures tested for immune reactivity and all epitopes were mapped to 10 reference HCV polyproteins and the best match identified (see methods). A unique molecular structure is defined by having been tested in at least one experimental assay in the original report. Using a multiple sequence alignment of the 10 polyproteins to the reference strain H77, a starting and ending position for each epitope in H77 was assigned. This simple operation allowed organizing and visualizing >98% of different unique molecular structures tested and reported in the literature.

The results of this process are illustrated in **Table 4**, where all structures containing or overlapping with the well-known HLA class II restricted epitope region NS3 (1248–1261) [10] are shown. For each structure, the IEDB epitope ID number, the sequence and the starting and ending positions are tabulated, along with number of positive assays and the number of total reported assays is shown. To date, more than 30 different structures have been reported for this region. As also shown in Table 4, each was tested in a different number of individuals and associated with different reported frequencies of positive responses.

**Table 4:** Response frequency data for immunodominant CD4^+^ T cell epitope NS3 (1248–1261)

Epitope Sequence	Position	Responders	Tested	RFscore
AYAAQGYKVLVLNPS	1243–1257	1	10	0.0±0.10
AYAAQGYKVLVLNPSVAA	1243–1260	12	22	**0.39±0.16**
YAAKGYKVLVLNPSVAAT	1244–1261	0	1	0.0±0.00
YAAQGYKVLVLNPSVAAT	1244–1261	0	2	0.0±0.00
AAQGYKVLVLNPSVA	1245–1259	0	1	0.0±0.00
AQGYKVLVLNPSVAA	1246–1260	1	1	0.0±1.00
QGYKVLVLNPSVAAT	1247–1261	1	10	0.0±0.10
QGYKVLVLNPSVAATLGFGA	1247–1266	1	1	0.0±1.00
GYKVLVLNPSVAAT	1248–1261	38	54	**0.59±0.11**
GYKVLVLNPSVAATL	1248–1262	2	2	**0.29±0.71**
GYKVLVLNPSVAATLGFGAY	1248–1267	1	1	0.0±1.00
YKVLVLNPSVAATLG	1249–1263	0	1	0.0±0.00
KVLVLNPSVAATLGF	1250–1264	12	12	**0.71±0.29**
VLVLNPSVAATLGFG	1251–1265	1	10	0.0±0.10
VLVLNPSVAATLGFGAYM	1251–1268	2	3	**0.2±0.47**
VLVLNPSVAATLGFGAYMSK	1251–1270	0	46	0.0±0.00
VLNPSVAATLGFGAY	1253–1267	0	1	0.0±0.00
NPSVAATLGFGAYMS	1255–1269	0	10	0.0±0.00
SVAATLGFGAYMSKA	1257–1271	0	1	0.0±0.00
VAATLGFGAYMSKAHGID	1258–1275	0	1	0.0±0.00
VAATLGFGAYMSKAHGVD	1258–1275	2	2	**0.29±0.71**
AATLGFGAYMSKAHG	1259–1273	0	10	0.0±0.00
TLGFGAYMSKAHGID	1261–1275	0	1	0.0±0.00

A larger region was chosen in order to allow for visualization of reactivity upstream and downstream of the known epitope.

### Combined Response Frequency scores allow visualization of epitope data

To identify the most well-studied and frequently recognized epitopes we derived a “response frequency score”, reflecting the overall frequency of recognition of epitope structures containing a given residue. A simple approach to measure response frequency would be to identify the number of individuals positive for a given epitope. However, this is not desirable. For example, recognition of a given peptide in 5 out of 100 individuals is quite different from recognition of a given structure in 5 out of 5 subjects, even though the absolute number of donors recognizing the two structures is the same. Conversely, to simply compile frequencies of recognition is also not desirable. This is because an epitope recognized in 1 out 1 donors tested would be assigned a higher frequency than another recognized in 99 out of 100 donors tested, even though the latter data is much more likely to indicate wide recognition in a broad population.

To address these issues we developed a response frequency score (RFscore) that estimates recognition frequency, conservatively weighted to factor-in statistical significance. For a given epitope, the un-weighted response frequency is calculated on the basis of the number of individuals tested and the number of individuals in which this test was positive. Recognition of a given epitope in 100% of the cases it was tested will given a frequency of 1.0, and complete lack of recognition would correspond to a frequency of 0.0. The RFscore is then calculated as (number responded – number responded∧0.5)/number tested. The square root is a correction factor, approximating one standard deviation for the number of responding donors. This gives a higher score to epitopes studied with larger sample sizes. For reference, an epitope positive in 10/10 donors will yield an RFscore of (10–10∧0.5)/10 = 0.9, and an epitope positive in 100/100 donors tested will have an RFscore of 0.98. Importantly, this formula can also be applied to structures that were negative in all assays and donors tested.

The tabular representation presented in Table 4, organizes the data in a consistent and more comparable format, but is still cumbersome, and does not allow for the integration of all information related to a given epitope/region. To meet this goal, we noted that the formula and process described above could be applied not only to individual epitopes, but also to individual residues. In this case, all epitope structures containing a given residue are collected, the total numbers of responding donors summed, and a combined response RFscore is calculated. The above method plots data accumulated for the recognition of linear peptides only and therefore is not readily applicable to discontinuous antibody epitopes.

**Figure 2:**
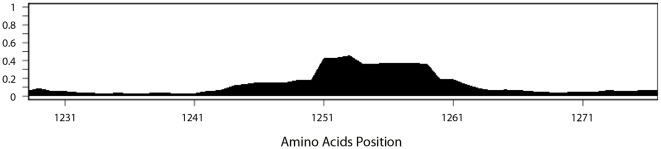
Response frequency score data for NS3 (1248–1261) mapped onto reference genome. Data represent epitope immunoreactivity for a region on the NS3 protein (aa1230–1275) containing the immunodominant epitope has been mapped onto the reference genome HCV strain H77. A larger region was chosen in order to allow for visualization of reactivity upstream and downstream of the known epitope. RFscores (frequency values in the 0–1 range) are shown on the Y-axis and the location on the HCV polyprotein in amino acid is presented on the X-axis. User inputs include the ‘start’ and ‘end’ position of the region of interest, the minimum and maximum axis value and the RFscore cutoff point to adjust stringency of data returned.

**Figure 2** provides an example of this process for the NS3 (1248–1261) epitope. Plotting the RFscores for the region including the epitope allows visualization of the epitope boundaries and response rates, based on the weighted combination of all available experimental data.

### Immune reactivity maps for HCV structural and non- structural proteins

Next, epitope maps for each individual protein were analyzed. RFscores for T epitopes are typically less for antibody epitopes, because even highly dominant T cell epitopes are only positive in individuals expressing the relevant restricting MHC molecules. To standardize the data, a red line that represents the average responses of T cell and antibody epitopes is shown in the figures. Averages RF values for all antibody and T cell responses were just under 0.4 and 0.2 (corresponding to percent values of 40% and 20%), respectively. The y-axis was also adjusted differently in order to better visualize the data.

**Figure 3:**
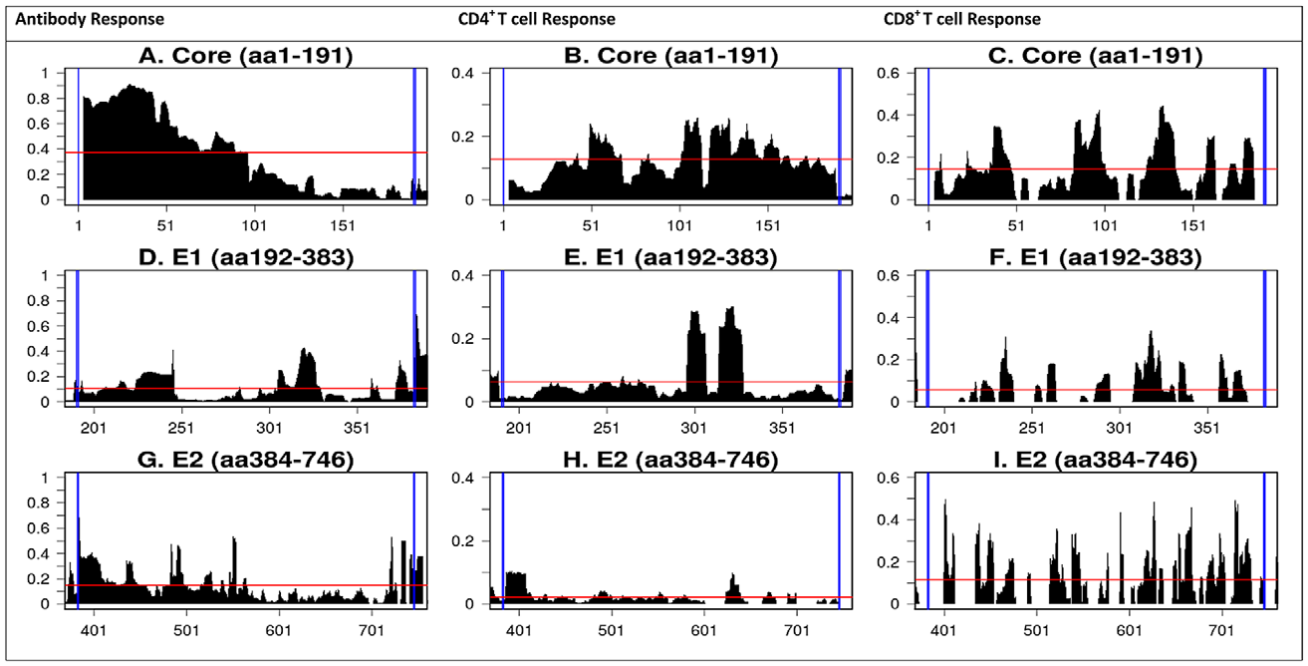
Detailed reactivity maps for HCV structural proteins. Data represent individual RFscores for antibody, CD4^+^ and CD8^+^ T cell responses plotted for each antigen translated from the HCV H77 reference polyprotein: A–C = core; D–F = E1; G–I = E2. The red line denotes average frequency for all antibody, CD4 and CD8 T cell responses. Note: Data displayed for antibody reactivity only include linear epitopes. Note: decimal values for RF scores on the Y-axis are converted to percent in the text.

The RFscores of individual HCV proteins (core, E1 and E2) is shown in **Figure 3**. For the core protein, the greatest antibody response frequencies (**Figure 3A**) were in the N-terminus with greater than 0.8 response frequency between aa1–50. In contrast, T cell responses (**Figure 3B, C**) were distributed along the length of the protein, with slightly more CD4 activity clustered toward the C-terminus (aa100–150). Antibody reactivity to E1 was mainly distributed over aa220–240, aa300–330, (RFscores 0.2–0.4 and aa370–383 (RFscore 0.4, **Figure 3D**). Much of the CD4 and CD8 T cell reactivity was found in aa300–340 (**Figure 3E, F**), and additionally in aa230–370 for CD8 responses. For E2, antibody reactivity was also highest in the N-terminal region (aa384–550), and maximal between residues 390–410. Lower reactivity was observed between residues 450–500 (0.25–0.4). Clusters of reactivity were also observed around aa540–550 (0.4–0.5 and near the C-terminus, aa720–746 (0.4–0.5, **Figure 3G**). E2 CD4 T cell reactivity was low (**Figure 3H**), whereas significant CD8 reactivity was apparent along the entire length of E2 (0.3–0.4) (**Figure 3I**). Reactivity for p7 was negligible (data not shown).

**Figure 4:**
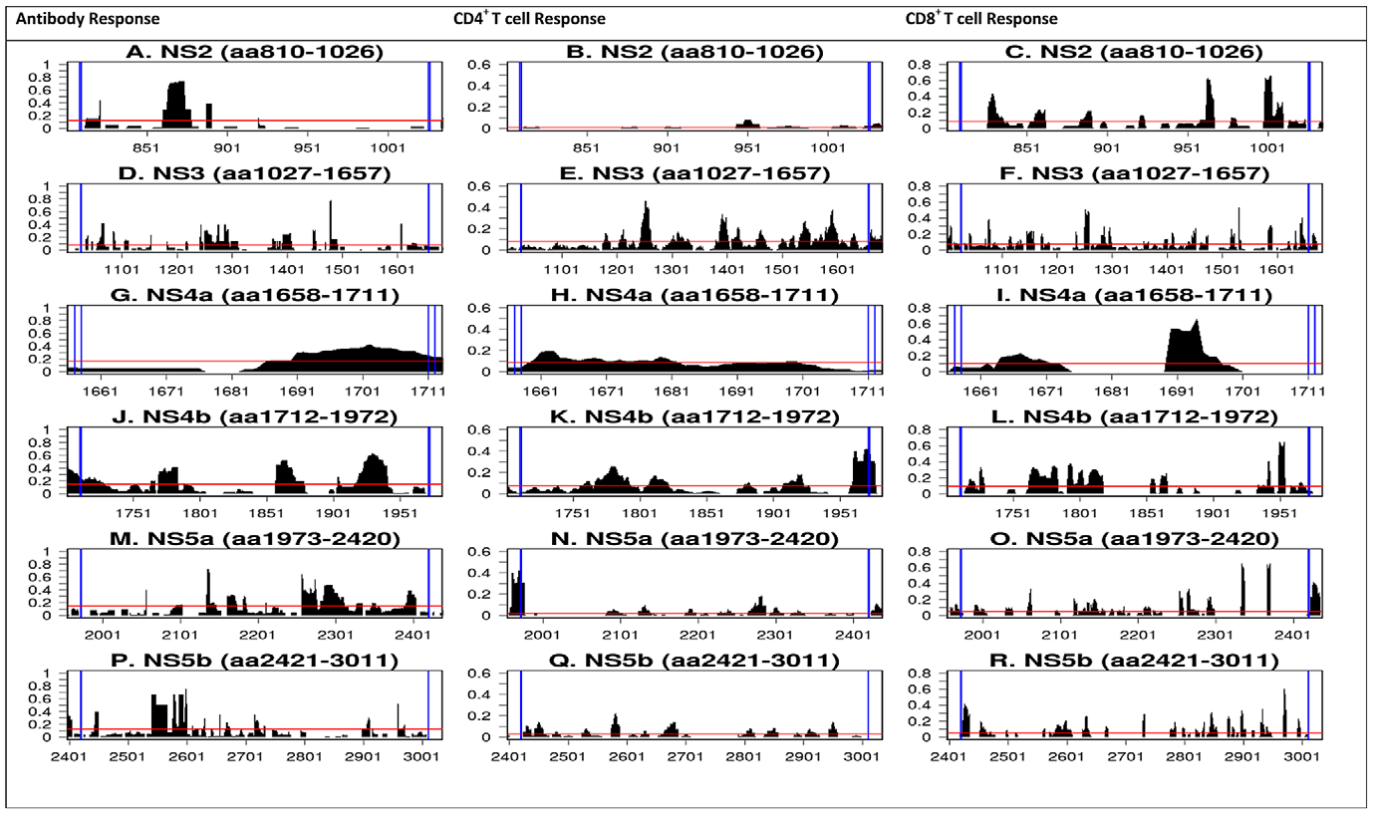
Detailed reactivity maps for HCV non-structural proteins. Data represent individual RFscores for antibody, CD4^+^ and CD8^+^ T cell responses plotted for each antigen translated from the HCV H77 reference polyprotein: A–C = NS2; D–F = NS3; G–I = NS4a; J–L = NS4b; M–O = NS5a; P–R = NS5b. The red line denotes average frequency for all antibody, CD4 and CD8 T cell responses. Note: decimal values for RF scores on the Y-axis are converted to percent in the text.

NS2 antibody reactivity is clustered between residues 860–880, with RFscores approaching 0.75 (**Figure 4A**). CD4 T cell RFscores for NS2 were low (<0.1, **Figure 4B**). By contrast, strong CD8 reactivity was observed in the N-terminus (aa810–860), as well as in the C-terminus (aa960–970 and aa1001–1010) (**Figure 4C**). In the case of NS3, a sharp peak of antibody reactivity was located at aa1460, with RFscores in the 0.75–0.8 range (**Figure 4D**). CD4 reactivity was low in the N-terminal region, and had several peaks of reactivity in the second half of the protein. By contrast, CD8 T cell epitope reactivity is high and distributed along its entire length (**Figure 4E, F**). Interestingly, the region around aa1250 is reactive for all three B, CD4 and CD8 responses.

Antibody reactivity for NS4a and NS4b were fairly high (**Figures 4G, J**). The region between the C-terminus of NS4a and the N-terminus of NS4b was fairly reactive (aa1685–1740; RFscores of 0.2–0.4). Notable frequencies for NS4b are also observed in three additional regions (aa1760–1780, aa1860–1880 and 1900–1950) with RFscores of 0.4, 0.5 and 0.6, respectively (**Figure 4J**). CD4 reactivities for NS4a are low, while CD8 reactivity is strong and concentrated around residues 1685–1700 (**Figure 4H, I**). For NS4b, CD4 and CD8 T cell response share a cluster between aa1760–1825 (also present in antibody response) and another significant CD4/CD8 reactivity cluster between aa1940–1950 (**Figures 4K, L**).

Finally, for the NS5a protein there is a sharp contrast in reactivity for antibody and T cell responses (**Figures 4M–O**). Clusters of positive RFscores are focused on the C-terminus (0.4) and in a short region in the center (∼aa2130; RFscores of about 0.6) for antibody responses. By contrast, low CD4 and CD8 reactivity was observed with the exception of CD4 responses at the very C-terminus, and two strong peaks of CD8 reactivity between aa2301–2401. NS5b shows one major cluster of antibody activity, focused mostly in the N-terminal region (aa2550–2600), with RFscores in the 0.4–0.7 range (**Figure 4P**). By contrast, CD4 and CD8 responses are dispersed along the length of the protein.

The data presented above suggest that little correlation exists in general between the HCV protein regions recognized by antibody, CD4 and CD8 responses. This point was addressed by correlating RFscores for the three responses over the entire length of the polyprotein. We found that none of the three variables correlated with each other, perhaps consistent with the different mechanisms underlining generating of immune responses mediated by antibodies, CD4 and CD8 responses (Pearson correlation R values ranged from 0.088 to 0.109). In conclusion the analysis presented above provides a detailed map of HCV-specific immune reactivity, and suggests that different HCV regions are independently targeted by immune responses.

### Correlation between epitope reactivity and the physical/functional properties of HCV proteins

**Figure 5:**
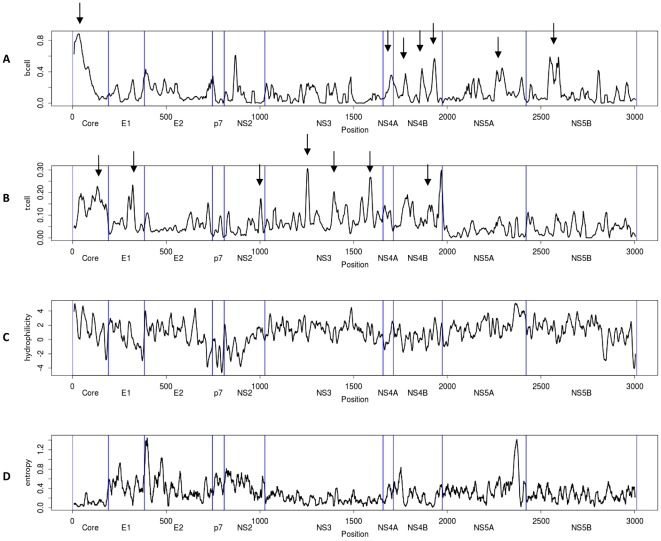
Analysis of immune responsiveness versus physical properties of HCV polyprotein. A) Antibody response (RF) scores plotted along the length of the HCV H77 polyprotein, B) T cell response frequency (RF) scores plotted along the length of the HCV H77 polyprotein, C) Hydrophilicty data calculated for the HCV H77 polyprotein using the Parker method, and D) Entropy scores calculated for all HCV sequence data available from the Los Alamos Lab HCV database and plotted along the entire ployprotein. Black arrows indicate regions of high imunoreactivity and low entropy.

Next, we examined possible correlations between the RFscores for antibody and T cell data, and the overall physical properties of HCV antigens. Using the Parker hydrophilicity prediction algorithm located on the IEDB antibody epitope prediction Tools page, we generated a hydrophilicity map for the HCV H77 genome. Overall antibody and T cell response frequency plots versus hydrophilicity are presented in **Figure 5**. We found a positive correlation between antibody response frequency and hydrophilicity, (p = 4.6 E-06), consistent with earlier reports [11]–[14] suggesting that hydrophilicity correlates with surface accessibility and antibody binding regions.

In terms of overall antibody reactivity, many of the dominant activities could be in fact be traced to exposed areas of the various HCV proteins. The dominant reactivity with the first 100 residues of the core protein may reflect that domain I is hydrophilic whereas domains II and III are hydrophobic and buried within the membrane [15]–[17].

Several studies have analyzed immune reactivity to the E1E2 complex [18]–[20]. Indeed, both monoclonal and polyclonal antibodies have been found to target linear, as well as conformational epitopes, within the E2 region, and these have been shown to mediate virus neutralization, preventing entry of the virus through blocking E2 binding to the host's CD81 co-receptor. NS2 is a non-glycosylated transmembrane protein [21], [22,], rapidly degraded after cleavage from NS3 [23]. Given the intensity of the antibody reactivity at aa860–880, this region maybe more stable and/or exposed. Diffuse antibody reactivity is observed for NS3. Possibly because of its localization at the ER membrane [24]–[26], and/or due to difficulty in preparing NS4A antigen, relatively low epitope reactivity was detected for NS4a. The C-terminus, however, contains acidic residues that can fold into an alpha helix and interact with the viral replicase and is characterized by a high density of B cell reactivity and high RFscores. [27]. NS4b is a 261 residue integral membrane protein that localizes to the ER, characterized by at least four transmembrane domains, [28]–[32], which might explain the pattern of four alternating clusters of B cell responses observed for this protein.

Interestingly, at the level of T cell responses we detected a negative correlation between T cell reactivity and hydrophilicity (p = 6.47E-09), suggesting that opposite structural features correlate with antibody and T cell recognition. The fact that hydrophobicity correlates with T cell antigen sites may be reflective of the tendency for MHC anchor residues to be hydrophobic. In contrast to what was observed with antibody responses, the epitopes targeted by T cell responses (both CD4 and CD8) are in general derived from the entire length of HCV polyprotein, with no particular correlation with the structures of the various HCV proteins. This is not unexpected because T cell reactivity and immune dominance are not dependent on folded protein structure, but rather from other variables such as protein abundance, rates of protein synthesis, cellular processing, MHC binding and TCR repertoire [33].

### Correlation between epitope reactivity and HCV sequence variability

Immune pressure can result in high sequence variability of pathogen sequences. This is particularly true for RNA viruses that mutate rapidly, such as HIV, SIV, influenza and HCV [34]–[36]. Highly variable regions would be expected to be associated with low RFscores. This is because different patients and patient subpopulations may be infected by, and responding to, different variants/quasispecies and these variants might be at least in part immunologically non-crossreactive. This would result in each individual variant being recognized with lower overall frequencies than more conserved ones. To examine this issue in more detail, we calculated entropy scores for all available HCV sequence data using the data and tools on the HCV sequence database maintained by Los Alamos National Laboratory (http://hcv.lanl.gov/content/sequence/HCV/ToolsOutline.html, **Figure 5D**) to provide a standardized measure of inter-sequence variability. Entropy was relatively constant along the length of the HCV polyprotein, with the exception of two prominent peaks corresponding roughly with the hypervariable region 1 (HVR1) within the E2 region and the PKR-binding domain (PKRbD) comprising the interferon sensitivity determining region (ISDR) within the NS5A protein. When entropy scores were correlated with T cell and antibody RFscores, both B and T cell reactivity were negatively correlated with entropy (p = 1.56E-02 and p = 8.69E-11, respectively). Thus as expected, conserved regions are more likely to be broadly recognized. Of particular interest, the analysis revealed several regions of low entropy and high immunoreactivity, marked by arrows in Figure 5. These regions may be of interest in the context of development and testing of HCV vaccination strategies targeting T cell responses. Given the historical prominence of the HCV 1 genotype in early studies, we thought it would also be interesting to investigate the relationship between entropy and immune reactivity for this genotype. When the analysis is restricted to epitopes known to occur in only HCV 1 and entropy is restricted to genotype 1 sequences we found that similarly, epitopes with high RFscores tend to have low entropy score (data not shown).

### Biological correlates of immune reactivity

**Figure 6:**
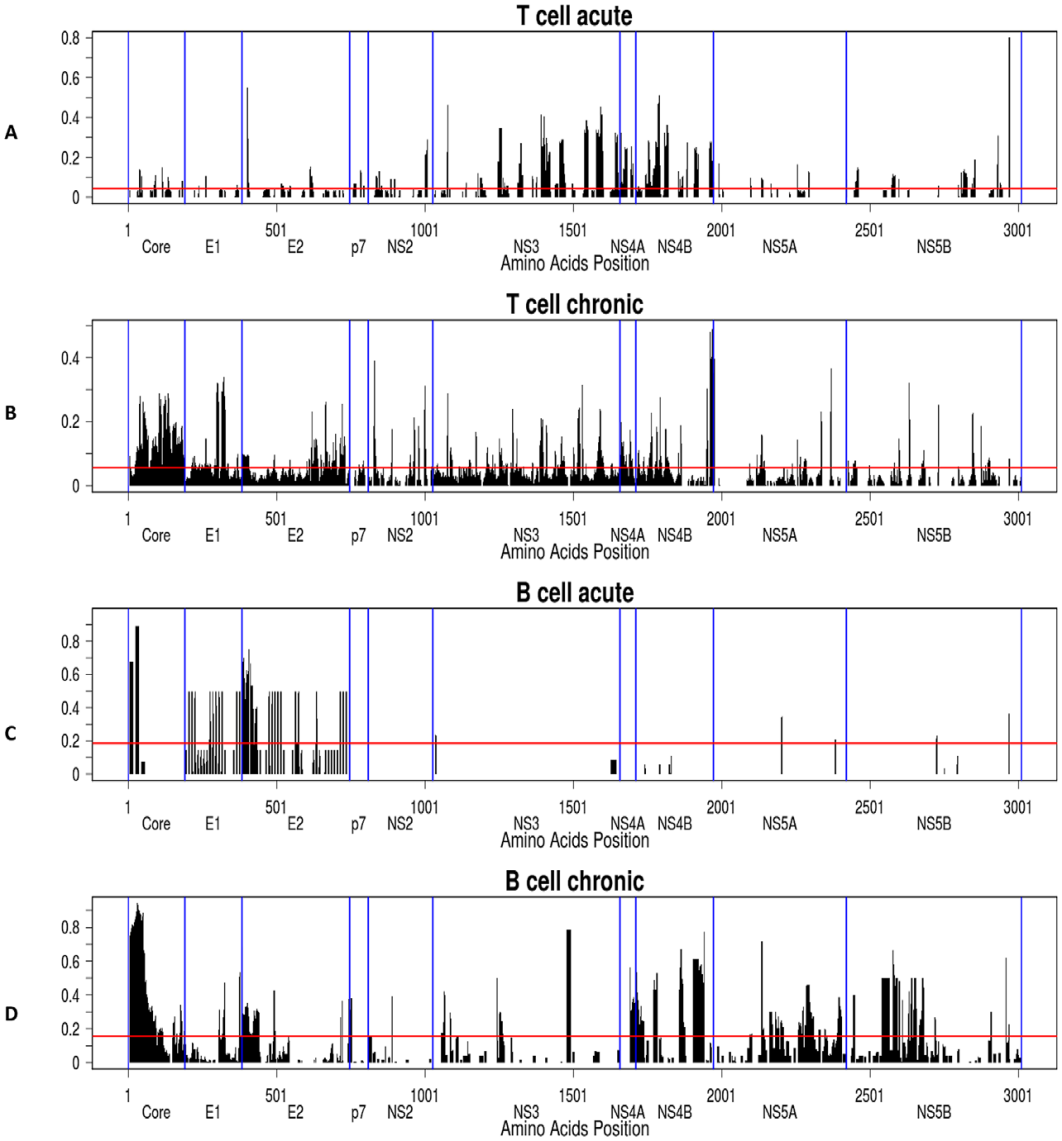
Human response frequency scores in acute versus chronic HCV infection. Data represent RFscores plotted along the length of the HCV H77 reference polyprotein for epitope defined in human disease. A) T cells responses for acute infection; B) T cells responses for chronic infection; C) Antibody responses for acute infection; D) Antibody responses for chronic infection. The red line denotes average frequency for all responses.

It is important to note that because acute HCV infection is typically asymptomatic, chronic HCV has been more thoroughly studied [37]. Here, we queried for all records in which the disease status of the host was identified in the patient history, according to the specific criteria defined in each paper by the respective authors. A total of 323 epitopes have been reported for acute disease, and a total of 1,871 epitopes for chronic disease. In terms of T cell responses, we observed an early robust response to NS3 and NS4 (mostly CD4; data not shown), which was maintained at a lower level during chronic disease, the appearance of reactivity to core, E1 and E2 in chronic disease and the relative lack of activity to NS5 (**Figure 6**). These results should be interpreted with caution, because early epitope mapping studies were performed primarily using NS3 and NS4, the recognized epitopes were then targeted by others in subsequent studies. Testing overlapping peptides that cover the entire HCV polyprotein [38] reveals little or no predominance of NS3/4 responses and rather strong NS5-specific responses, suggesting that a major difference between acute and chronic T cell responses is the vigor of the response, rather than the specificity. Antibody reactivity was minimal in acute disease, with the exception of the N-terminus of the core protein, E1 and E2. By contrast, and as expected, robust antibody reactivity was observed for chronic disease. Prominent antigens included core, E1 and E2, as well as NS4 and NS5. Interestingly, in the case of NS3, few epitopes were described in the literature therefore resulting into low RFscores for this antigen. Considering the known immunodominance of this antigen [39], [40] the data suggests that the true immunodominant epitopes associated with this antigen might yet have to be described and characterized. Specifically, NS3 was one of the earliest antigens developed for HCV diagnosis. The commercial antigen c33 encodes the entire NS3, but not short peptides.

The relative lack of antibody response during acute infection and subsequent emerging reactivity during chronic infection is not unexpected due to the short duration of acute infection and normal kinetics of appearance of significant antiviral antibody titers.

HCV-specific antibodies are first detectable in acute infection and coincide with elevation in liver enzymes. Titers of HCV-specific antibodies, including neutralizing antibodies against multiple strains increase during chronic infection. Therefore it is thought that whereas T cell responses exert selection pressure mostly in the acute phase, antibodies exert selection pressure mostly in the chronic phase [37]. Accordingly we examined the data with respect to the number of assays demonstrating neutralizing versus non-neutralizing antibody responses. We found that the vast majority of reactivity was determined using non-neutralizing, direct-binding assays. Of the more than 7000 antibody assays reported to date for HCV, 3800 are associated with positive results, and only 84 involve neutralizing assays. For those assays, epitope specificities derived from the E2, a well-established target of neutralizing antibodies in human infection [41]–[43], were predominant (**Table 5**). Low numbers of neutralizing epitopes were also reported for E1 (6) and NS5b (4). The reports of neutralizing epitopes is surprising since NS5b is not known to be present on HCV mature viruses. Furthermore, we noted that all discontinuous epitopes characterized so far were derived for E2, and of these 19 of 23 were defined in the context of neutralization. These data underscore the need to further characterize the HCV epitopes associated with neutralizing activity.

**Table 5:** Characterization of Humoral Response by Antigen

HCV Antigen	Neutralizing assay for linear epitopes	Other antibody responseassay for linear epitopes	Neutralizing assay for discontinuous epitopes	Other antibody response assay for discontinuous epitopes
Core	0	396	0	0
E1	6	321	0	0
E2	50	1,264	19	4
p7	0	12	0	0
NS2	0	39	0	0
NS3	0	156	0	0
NS4a	0	349	0	0
NS4b	0	474	0	0
NS5a	0	385	0	0
NS5b	4	435	0	0

Antibody immunoreactivity was analyzed by *assay type* for each HCV antigen. Two categories are presented: neutralizing assays (standard neutralization assays or any inhibition of antigen activity assay) and other antibody response assays (ELISA, FIA, ELISPOT, immunoblot, RIA, SPR, Immunopanning, mass spec, challenge assay, etc.). Data shown are the total number of assays, not epitopes.

We next investigated the influence of glycosylation on immune reactivity, since it has been reported that these moieties are involved in the negative modulation of virus neutralization [18]. The E2 protein contains 11 glycosylation sites modeled throughout the protein [44]. The average RFscores for glycosylated sites were compared with the average RFscores of all non-glycosylated sites in the E2 protein. No significant difference was found for antibody responses. However, we found a significant negative impact on T cell reactivity (p = 8.1e-07). Whether this negative modulation occurs at the level of antigen processing or at the level of T cell recognition cannot be determined by this study.

## Discussion

Herein, we report the first meta-analysis of HCV immune epitope data. The data is derived from hundreds of different published reports and relates to thousands of different molecular structures, analyzed in over 18,000 different assays, all curated within the IEDB analysis resource.

The inventory of HCV epitope data revealed that while most records are derived as expected from humans and chimpanzees, a surprising large number of records (about 12%) were also derived from murine systems. Furthermore, the inventory also revealed substantial imbalances in coverage of different HCV serotypes by the different types of effector responses (antibodies, CD4 and CD8), thus suggesting areas that require generation of additional data.

The large number of records required the development of a method to combine and display immune epitope data from a number of different scientific reports. The approach was used to analyze the HCV epitope data, determining which antigens are dominant for the different response types, and draw detailed maps of reactivity for the various different antigens. CD4, CD8 and antibody reactivity were not correlated with each other suggesting that each is governed by distinct variables. Taking advantage of the large data set, we were able to show a positive correlation between antibody epitope reactivity and hydrophilicity and a negative correlation for T cell epitope reactivity. These results highlight, as previously pointed out in the case of immune recognition of pox viruses [45] that different structural features influence and dictate antibody and T cell recognition.

Epitope reactivity was also correlated with sequence variability. Highly variable regions were associated with low frequency of reactivity. This is because each of the immunologically non-crossreactive variants resulting from immune escape can only be present and hence recognized in a fraction of the patient population. This results in each individual variant being recognized with lower overall frequencies than more conserved ones. One important caveat should be considered in reviewing these data, namely that the vast majority of published studies were conducted with HCV peptides/antigens that do not reflect the sequence of the infecting virus, which in many cases is unknown. This may especially affect the score for highly variable regions; such as HCV envelope sequences because if a study tests a peptide that does not match the infecting strain, the result may be negative even though a peptide from the same region of the infecting strain would be positive. In contrast, other regions such as HCV core are highly conserved and therefore, use of HCV genotype 1a peptides in immune response analyses may yield more hits. Regardless of these considerations, our analysis also revealed a number of regions associated with high immune reactivity and low variability, of potential interest in the context of vaccine development.

Herein, all epitope data was eventually mapped to a single reference genome. In this respect our approach is of general applicability since it can be applied to datasets derived from any organism for which a reference genome is available. Next, our approach allowed calculating for each structure the frequency of individuals for which a positive response was detected over the total tested. Thus, the approach is highly innovative, as for the first time allows taking into account also negative results. By contrast, most immunological databases only report positive data, thus making it difficult to establish the broad significance of well-established versus anecdotal data.

However, the results obtained must be interpreted with important caveats in mind. First, this analysis can obviously only consider published/reported data, as curated in the IEDB. Lack of positive (or negative) data relating to a given structure cannot be taken to imply that the structure is negative, but simply implies that data related to the structure are not available. Whether the region has been analyzed and found to be negative, but the negative data was not published cannot be determined by a meta-analysis. An unbiased approach of testing T cell responses with overlapping peptides that cover the entire HCV polyprotein was widely used only after the first decade or so of HCV immunology research. During the first 10–15 years of study, many investigators used single peptides that had been selected because they contained specific HLA-binding motif, had a high HLA binding affinity and were then shown to be recognized by T cells. For example, most early studies used predicted HLA-A2 peptides in in-vitro stimulation assays, whereas now many studies are performed directly ex vivo, using peptide libraries targeting overlapping, as well as previously described epitopes. After the first epitopes were identified, many studies were conducted with those epitopes, and as a consequence, certain regions were more frequently studied than others. Second, in the current approach negative data is plotted as “zero” regardless whether the zero value is derived from a single observation or negative results have been established in a large number of observations. Thus, the method is best suited to highlight which positive data are most reliable. There are also significant qualitative differences among studies with regard to how positive responses were defined and/or confirmed. We are currently exploring different visualization methods, to address this important issue. Third, the RF method like any other method plotting discrete variables along a sequence is not suited to visualize discontinuous epitopes. Here as well, we are currently exploring different visualization methods, to address this important issue.

It is also important and relevant to address the issue of the reagents. Historically, most reagents are based on HCV genotype 1, however the genotype of the infecting virus in human subjects is often heterogonous and/or the subject may have been infected with more than one strain. As a result, the cumulative data will miss many responses because of the impossibility of using autologous sequence reagents. Moreover, it is possible that the data will reflect responses that are actually not associated with the currently infecting genotype.

Our analysis allowed investigation of issues related to the biological relevance of immune responses. First, comparison of data derived from acute and chronic infection revealed fine differences in specificity of T cell and antibody responses, though due to the complex nature of this disease, the data must be interpreted with the understanding that there are very different definitions in the immunological literature regarding what constitutes acute infection. In some instances, studies include subjects up to 2 years post-infection for analysis, despite the fact that after 6 months the outcome has been firmly established in most cases and immune responses change dramatically in the first year. Different methodologies may also play a role, in that older studies tended to focus on testing subjects with persistent chronic infection, whereas, chronic infection. Large scale studies including subjects with acute infection have only been performed recently using the newer ex-vivo methods. Second, the analysis revealed that, unexpectedly, glycosylation seem to interfere with T cell recognition, but not antibody responses. Third, the data revealed a need for far more detailed definition of epitopes recognized by neutralizing antibodies.

The methodology described herein provides the ability to combine the data from the literature in a format which is comprehensive, yet intuitive. Ultimately, it is our goal to implement this method as a general tool on the IEDB website, to provide the larger scientific community with the functionality to easily analyze immune epitope data.
